# Support practices by an interdisciplinary team in a palliative-care unit for relatives of patients in agonal phase

**DOI:** 10.1186/s12904-020-00680-4

**Published:** 2020-11-19

**Authors:** M. Mélin, H. Amieva, M. Frasca, C. Ouvrard, V. Berger, H. Hoarau, C. Roumiguière, B. Paternostre, N. Stadelmaier, N. Raoux, V. Bergua, B. Burucoa

**Affiliations:** 1 Palliative Care Service, Universitary Hospital Center Bordeaux, 1, rue Jean Burguet, 33075 Bordeaux, France; 2 Inserm 1219 Psycho-epidemiology of Aging and Chronic Diseases - Population Health Research Center-ISPED-University Bordeaux, Bordeaux, France; 3 URISH, Universitary Hospital Center Bordeaux, Bordeaux, France; 4 Bergonié Institute, Bordeaux, France

**Keywords:** Agony, Support practices, Relatives, Interdisciplinarity, Palliative care

## Abstract

**Background:**

In the absence of extant recommendations, the aim of this study was to formalise support practices used by an interdisciplinary team in a palliative-care unit (PCU) for the relatives of patients in the agonal phase preceding death. The secondary objective was to understand the expectations of relatives during this phase in terms of the support provided by professionals and volunteers.

**Methods:**

Thirty-two people took part in this study; all were interviewed through focus groups (FGs). Each FG comprised one category of individuals working in the PCU: nurses, care- assistants, doctors, psychologists, other professionals, palliative-care volunteers, and relatives. Groups were surveyed using an interview guide, and the interviews were recorded and transcribed to enable identification and characterization of all practices. Care practices were classified into four categories: current consensual practices (i.e. performed by all team members), occasional consensual practices, non-consensual practices (performed by one or a few participants), and practices to be developed.

**Results:**

In total, 215 practices were mentioned by professionals and palliative-care volunteers: 150 current consensual practices, 48 occasional consensual practices, 1 non-consensual practice, 16 practices yet to be developed, and 29 practices for relatives. Many practices were mentioned by different categories of participants; thus, after cross-checking, the number of practices decreased from 215 to 52. A list of practices deemed desirable by all was drawn up and then validated by the entire interprofessional team. These practices were organised around four themes: providing care and ensuring comfort; communicating, informing, and explaining; interacting; and mobilising interdisciplinary skills.

**Conclusions:**

These results underline the importance of the quality of care provided to patients, the attention given to the relatives themselves, and they highlight the importance of the helping relationship. Following this study, which established a list of varied practices aimed at supporting the relatives of patients in agonal phase, it will be important to set up a broader study seeking to establish a consensus on these practices with an interprofessional group of experts from other PCUs using broad surveys and an adapted methodology. Such studies will make it possible to develop training modules for teams working with relatives.

**Supplementary Information:**

The online version contains supplementary material available at 10.1186/s12904-020-00680-4.

## Background

The 1990 World Health Organization (WHO) definition of palliative care, the French law on access to palliative care (Law 99–477), and French ministerial recommendations between 2004 and 2018 emphasise the importance of ‘supporting the patient’s relatives’, particularly in preventing difficult or pathological bereavements [1–3]. The support of relatives qualified as ‘caregivers’ is a major focus of work due to the psychosocial impacts of progressive disease on the patient, his or her entourage, their relationships and their shared goals [4]. This is a key element in enhancing the quality of patient care and support [5].

Most research on the needs and perceptions of family caregivers, their stress and fatigue levels and their satisfaction with care concludes that it is necessary to improve their education about the end of life and the support provided to them [6]. Family discussions, for example, can help to alleviate the frequently observed discrepancies between the sick person and his or her relatives regarding the clinical situation, the course of the disease and the patient’s vital prognosis [7]. Such discussions contribute to a decrease in physical and psychological symptoms in relatives, improvement in their quality of life and enhanced communication with professionals [6].

A recent multinational study of family carers in Belgium, Germany, Hungary, the Netherlands and the United Kingdom revealed that many teams did not pay sufficient attention to the needs of the carers, and did not provide proactive care and access to support [8]. Similarly, in a qualitative study in a rural district of central Norway, despite the relatively well-known positive effects of systematic carer support, carers reported that they did not receive such support [9].

An understanding of how to implement person-centred interventions in palliative and end of life care is lacking, particularly to support family carers. To address this, components of the Carer Support Needs Assessment Tool (CSNAT) intervention were investigated in one study [10]. In another study conducted in north-east England (2011–2014), the issues faced by carers of patients with cancer and advanced, progressive illness, in their last year of life, were explored and an alert system for use by non-specialist staff, known as the Carers’ Alert Thermometer, was developed [11].

Support for relatives seems even more critical in the terminal phase, particularly during the agonal phase. Commonly people associate agony with anguish and suffering. It, which is defined in the French dictionary (Larousse) as the ‘state of the human being in the period immediately before death, when the organism may appear to be struggling to stay alive’. The agonal phase is scientifically characterised by ‘the appearance of the first signs of decerebration and the alteration of neurovegetative regulatory functions‘, which is inevitably followed by death [12, 13]. If the symptoms are controlled, patients in this final stage of life may not show any signs of suffering. However, suffering is experienced and expressed by their relatives.

In this context, relatives, already suffering from the previous course of the disease, face a major change in communication patterns, body appearance and symptoms due to mechanisms that are often difficult to understand for families [14, 15]. At the moment of death, relatives are confronted with the reality of their mortality. This stage can be trying, or even unbearable, or pointless for relatives [16]. The experience is often perceived as difficult to endure psychologically and existentially by relatives and society. Thus, support for relatives before the death of a patient frequently poses complex problems. Recommendations for care during the very last days of life have been published [17–19], but none specifically addresses support for relatives. Moreover, there is great heterogeneity in practices between specialised and non-specialised teams, and even among professionals within the same unit.

The main objective of this study was to identify the practices used by healthcare professionals and palliative-care volunteers to support relatives during the agonal phase in a palliative care unit (PCU). The secondary objective was to understand the expectations of the relatives in terms of support.

## Methods

### Population

Participants were recruited from the PCU of Bordeaux University Hospital, which provides multidisciplinary support for relatives, as described in several previous studies [7, 12, 16]. All of the staff of the PCU, which opened in 1995, were invited to participate in this study. According to their availability, 25 staff involved at different levels in the support of relatives agreed to participate: three nurses, four care-assistants, five physicians, two psychologists, seven “individual professionals” (IPs) (psychometrician, secretary, socio-educational assistant, socio-aesthetician, health executive, physiotherapist, hospital service agent) and four palliative-care volunteers. The participants had between 3 months and 20 years of experience of working in a PCU (average of 8 and 3 years of experience among physicians and all other staff, respectively). In addition, seven relatives (the spouses of patients, aged between 40 and 70 years) were interviewed. They met the following criteria: accompanied the patient (including “basic body care”); a trusted by the patient; relative of a patient whose agonal phase had lasted more than 24 h and whose death had occurred at least 3 months before the study; and provided consent to participate in the study.

### Procedure

Information about the objectives and modalities of the research was presented at meetings and in a document distributed to all professionals in the unit and to the volunteer coordinator. Based on the volunteers’ schedules and the availability of each person, focus groups (FGs) were constructed to include various categories of participants.

Seven relatives meeting the inclusion criteria were contacted by telephone. After they agreed in principle, a document presenting the study was sent to them to obtain their consent to participate.

Verbal consent was obtained from all participating professionals and volunteers, in accordance with French law. Relatives confirmed their participation by mail. In France, this type of research does not require approval from a national ethics committee.

### Focus group method

Data collection was based on the FG method, a group interview technique for gathering information on a targeted topic and collecting field knowledge and a wide range of opinions [20]. The FGs were organized according to the category of the participants: physicians, nurses, care assistants, psychologists, volunteers and relatives. The aim of this distribution was to facilitate open expression and to improve the quality of the information. The IPs met at a multi-professional meeting. These FGs were led by two psychologists; one was from outside the service and the other was part of the team. Interview guides developed for this study were used for all data collection (Additional files 1 and 2), and exchanges during the FGs were audio-recorded. The study was carried out from December 2017 to February 2018.

During the FGs, each practice mentioned was presented, discussed and then classified. Some were classified as consensual among the group participants (i.e. practiced by all participants). These were further divided into practices applied routinely, those applied occasionally and those that were applied rarely but merited greater application. All other practices were classified as non-consensual (i.e. practiced by only one or a few participants).

Verbatim audio recordings and additional notes (taken by the psychologists) were collected during the FG sessions. Also, practices were discussed and classified on flipchart.

### Analysis strategy

#### Identification of practices by two judges

To avoid errors, the audio recordings of each FG session were transcribed in full. Two individuals who were not part of the study steering committee checked the written transcripts against the recordings. All identifying data were removed.

Based on this, and with the help of the notes and flipchart completed during the FG session, the two psychologists independently carried out manual identification (i.e. unassisted by software) and classified the practices (current consensual, occasional consensual, consensual to be developed, non-consensual). They then compared their results and discussed again any practices for which their classifications differed (only 5% of practices), resulting in a final list of practices and their classifications.

#### Validation/stabilisation of practices by all professionals in the unit

The practices thus defined according to categories of participants were collected and distributed in hard copy to the participants in the categories concerned, including both those who had and those who had not participated in a FG, asking them to validate these proposals or, if necessary, to propose amendments to the wording. The full list of practices classified as consensual was submitted to the entire professional team at a meeting and then in a printed form that could be annotated. During this process, some professionals included practices in their category that had already been named by other categories of participants, but no new practices emerged. The list of consensual practices was thus stabilised.

#### Thematization and synthesis of practices

All practices were grouped into major themes by the study steering committee and synthesised.

## Results

Codification of the basic transcripts resulted in a list of 215 practices, which were then collapsed into 52 practices. Categorisation of practices led to the identification of four themes.

Using the verbatim records from FGs of professionals and palliative-care volunteers, 215 practices were identified: 150 current consensual practices, 48 occasional consensual practices, 1 non-consensual practice and 16 practices to be developed (see Table 1).

**Table 1:** Number of practices by focus group (FG) and by category

FG/ Practices	Current consensual	Occasional consensual	Non-consensual	To be developed	Total
Nurses	21	2	0	5	**28**
Care assistants	12	6	0	1	**19**
Physicians	53	6	1	4	**64**
Psychologists	19	9	0	2	**30**
Individual professionals	25	16	0	3	**44**
Palliative-care volunteers	20	9	0	1	**30**
**Total**	**150**	**48**	**1**	**16**	**215**

The number of practices cited is variable according to the FG. The physicians mentioned the greatest number of practices (*n* = 64), and the care assistants mentioned the fewest practices (*n* = 19).

There were 214 thematic practices, as a non-consensual practice was excluded.

As stated above, the practices were organised into four themes: 1) providing care and ensuring comfort; 2) communicating, informing and explaining; 3) interacting; and 4) mobilising interdisciplinary skills (see Table 2).

**Table 2:** Number of consensual practices by FG and by theme

Professionals/Themes	Providing care and ensuring comfort	Communicating, informing, and explaining	Interacting	Mobilising interdisciplinary skills	Total
Nurses	9	8	5	6	**28**
Care assistants	8	4	5	2	**19**
Physicians	28	23	7	6	**64**
Psychologists	10	4	7	9	**30**
Individual professionals	7	9	11	16	**43**
Palliative-care volunteers	2	11	11	6	**30**
**Total**	**64**	**59**	**46**	**43**	**214**

Many practices were mentioned by multiple categories of participants. These practices were cross-referenced, reducing the number of practices from 214 to 52. The practices (Table 3 and Additional file 3) were taken directly from the transcripts. Table 3 presents the 52 practices classified according to theme. Additional file 3 presents practices in more detail.

**Table 3:** List of practices by theme

Themes		
**Providing care and ensuring comfort**	1. Provide patient care before the relatives enter the room, if necessary	2. Support requests for relatives to perform care or care-sharing (co-care) for the patient, depending on the relatives
	3. Take care of relatives through care given to the patient	4. Attend to physical needs
	5. Ensure the comfort of relatives in the unit	6. Propose a massage to relatives if trained to do so
	7. Propose an approach using relaxation, hypnosis, or eye movement desensitisation and reprocessing, depending on the situation and the psychologist’s training	8. Invite relatives to leave the room or to use the family room
	9. Offer to provide some respite time for the family	10. Allow family and friends to recreate a moment of intimacy with the sick person
	11. Psychologically prepare relatives for their entry into the room	12. Inquire about absent relatives
	13. Ensure that relatives are surrounded and supported by an entourage	14. Attend to children
	15. If necessary, grant a request for make-up for the patient after death	16. Conduct assistance interviews Personalise these in terms of objectives and content and in case of a request for euthanasia
	17. Inform relatives about what they will see in the room before entering; explain the medical devices and equipment once inside accompanying them to the room	18. Answer questions related to pain
	19. Explain the care, its impact on the patient’s well-being, and its continuation	20. Announce entry into agonic phase
	21. Help relatives to recognise the signs of agony that will appear	22. Explain the patient’s condition and visible symptoms
	23. Answer questions regarding the patient’s level of awareness of reality	24. Check whether the expectations of family members are being met
	25. Inform relatives that caregivers will be entering the room more often because the patient can no longer call them	26. Inform of the imminence of death
	27. Respect the relatives’ wishes concerning the announcement of the death	28. For relatives who wish to be present at the time of death, warn them that this may not be possible
	29. Give relatives an opportunity to indicate that they do not wish to be present at the time of death	30. Inform relatives that they can call whenever they want to, even at night
	31. Anticipate the steps that will need to be taken after death	32. Announce the death to relatives in person or by phone, provided that the nurse has received formal or informal training
	33. Make physical contact with loved ones (touch or be touched) as the situation arises	34. Receive the request for euthanasia
	35. Talk about something other than the situation	
**Communicating, informing, explaining** **Interacting**	36. Welcome and approach relatives; speak to them in the corridor if they are not familiar; show availability in a non-verbal way; establish a climate of trust	37. Propose listening times, a silent presence
	38. Propose a formal interview; in a dedicated space; with others who are close to the patient; include several professionals; in person or by telephone; post a sign to indicate that the room is in use; schedule the interview outside regular hours if necessary; especially in the case of a request for euthanasia	39. Defer non-urgent care if a close relatives visits
	40. Consider the patient’s socio-cultural and religious practices	41. Keep young children occupied during the visit
	42. Propose that relatives stay the night	43. Encourage family and friends to contact the doctors and members of the care staff
**Mobilising interdisciplinary skills**	44. Work in pairs such as nurse and nursing assistant	45. Propose a multi-professional interview
	46. Specifically include attending to young children during an interview with other professionals present, including the psychologist	47. Hand off tasks between peers
	48. Pass the patient care role on to other members of the PCU and to cultural representatives	49. Serve a third-party function between the team, family, and patient
	50. Design an interdisciplinary support project for relatives	51. Consider setting up a weekly meeting with relatives to discuss the general functioning of the PCU and to inform them of the team’s position on certain issues with the participation of caregivers and palliative-care volunteers
	52. Provide talking spaces	

The theme ‘Providing care, ensuring comfort’ comprised 16 types of practices (P1–P16). These included caring for the patient’s body, considered by the participants as involving the relatives (P1–P3) and attention paid to the physical well-being of relatives and their comfort (P4–P7). Other practices were related to the time devoted to relatives and the spaces where they could engage (P8–P11) and to the caregivers’ attention to the extended family and the entourage of the relatives (P12–P15). Maintenance as relational care was mentioned by all FGs of professionals except for the care assistants (P16). The nurse and care-assistant groups emphasised physical and comfort care, whereas physicians and psychologists emphasised relational care.

The theme ‘Communicating, informing and explaining’ included 19 types of practices (P17–P35). These practices were named primarily by physicians and palliative-care volunteers. This theme encompassed announcements (e.g. entry into the agonal phase, death prognosis or what has happened), answers to questions and descriptions of what was done during care. Caregivers, especially nurses, physicians and care-assistants, informed others about the stages marking the agonal phase and conveyed warnings in cases of increased severity (P17–P26). Some practices concerned the anticipation and announcement of death (P27–P31) and the time of death (P32).

Finally, other practices concerned physical contact with relatives (P34), being present and listening to relatives’ concerns (P34 and P35).

The theme ‘Interacting’ included nine types of practices (P36–P43). These practices were mentioned mainly by the palliative-care volunteers and the IPs. This theme comprises practices related to the setting up of a framework for exchanges with relatives, including contact with relatives, approaching relatives (P36; mentioned in all FGs) and proposing an informal or formal interview with the aim of deepening support (P37 and P38; mentioned in four FGs and by four IPs). Some practices were related to the personalisation of care (P39–P43), and some practices cited by the nurses addressed the adaptation of care to the relatives’ visits and to the patients’ socio-cultural and religious practices (P39 and P40).

The theme ‘Mobilising interdisciplinary skills’ included nine types of practices (P44–P52), mainly mentioned by IPs. These were practices such as acts carried out by two or three people (P44–P46), transitions when relaying tasks among peers (P47) or to other categories of professionals in the PCU (P48), a practice cited particularly by an IP, the function of a third party for the psychologist (P49), using a genosociogram or proposing meetings or “talking spaces” (P50–P52).

Relatives mentioned 29 practices: 13 consensual practices already implemented, 4 consensual practices to be developed, 4 non-consensual practices implemented and 8 non-consensual practices to be developed (Table 4). The 17 consensual practices were thematised and then cross-referenced with the 52 final practices described above.

**Table 4:** Number of practices identified by relatives

Relatives	Practices realised	Practices to be developed	Total
Consensual	13	4	**17**
Non-consensual	4	8	**12**
**Total**	**17**	**12**	**29**

Concerning the theme ‘Communicating, informing and explaining’ (eight types of practices), the value of information on the presence of volunteers and religious representatives (P48) and on the imminence of death (P26) and the possibility of talking about something other than the disease (P35) was noted.

The relatives mentioned the importance of preparing for the announcement of entry into the agonal phase (while being aware of the difficulty for doctors in determining it precisely) (P20) and the proposal to meet with a psychologist (P48), as well as other practices cited by professionals (P6, P21, P23 and P27).

For the theme ‘Providing care, ensuring comfort’ (six types of practices), they stressed the importance of professional caregivers’ invitation to take care of them (P9). Similarly, they cited proposals to provide care with a professional (P2), to use a family room (P8) and to have self-care time (P16), especially during the last days of life. They perceived certain care provided to the patient as also supporting themselves (P3).

For the theme ‘Interacting’ (three types of practices), the relatives mentioned the relevance of the following practices: the possibility of staying in the unit even at night (P42) and the invitation to ask for help from the caregivers (P43). They described the unit as ‘almost like a home’.

## Discussion

This exploratory study described support practices deemed desirable by an interprofessional PCU team, palliative-care volunteers and relatives of dying patients. Numerous and varied practices were described addressing care practices, attitudes and behaviours and testifying to the importance of interprofessional work.

In addition to individual practices, this study highlighted four major areas of practice in supporting relatives: practices designed to promote interaction, those designed to promote information-sharing and communication, those that provide care and comfort and those involving interdisciplinary cooperation. These results illustrate crucial concepts for supporting relatives in the field of palliative care.

### Therapeutic alliances and interdisciplinarity

All participants said that they needed to ‘approach relatives’, ask for exchanges with these people and show their availability (P36), especially during the agonal phase. By proposing formal and informal interviews, the caregivers helped relatives to mobilise necessary resources in the face of the ordeal of the agonal phase (P38). This reception served as an envelope for relatives in the pre-mourning period. They began to ‘become familiar with the prospect of death to be able to accept the difficult ordeal little by little, to gather the dying person’s last testimonies and messages, [...] and to support him or her in this final ordeal by giving him or her the maximum presence and affection’ [2]. Thus, the listening ability, availability, empathy and non-judgement of the interveners can facilitate the expression of those close to the dying person. Efforts to provide a climate of trust (P1) and the invitation for an exchange (P43) contributed to the creation of a therapeutic alliance, defined as a ‘mutual collaboration’ or ‘partnership’ between the patient and the therapist to achieve the objectives set [21, 22]. It also involves collaboration between the therapist and the relatives [23].

The theme ‘Interacting’ was important in the volunteers’ FG, which emphasised their inclination to offer specific support to relatives. Their place with the relatives was not defined by actions but by being present and listening. The IPs also made extensive reference to this theme. Interactions among professionals, palliative-care volunteers and relatives appear essential, given the physical and psychological morbidity of caregivers of patients in palliative situations (social isolation, accomplishment of many tasks), and the fact that they often feel ill-prepared for their role [24]. Their communication with the sick person is impaired and another stage in their relationship begins. Relatives said, ‘We are in a second state. We know we are going towards death’. They witnessed the patient’s loss, the alteration of his or her capacities and vigilance, relived the stages —through announcements, hopes, failures, anger and despair—and were confronted with their own vulnerability and their own death to come. Relatives questioned the meaning of this stage, of their presence and of their words when the dying person’s vigilance was impaired.

They expressed the hope that this period would not last long and their intense experience of facing the image of a degraded body. Their ambivalence was tangible, as they were torn between the desire to see the patient live and the desire for him or her to die without suffering.

The theme ‘Mobilising interdisciplinary skills’ was cited frequently by the IPs. The majority of this group work part-time in the unit, so a significant part of their activities involves liaising with their colleagues, participating in the transmission of information and informing themselves about care or steps taken in their area (P44, P48 and P50). Working in pairs seemed necessary to the participants to provide a dual view of situations (e.g. pain assessment; P44), and the term ‘transmission’ was used by all of them (P48). This act of ‘working together’ with coherence and cohesion requires knowledge of the profession and the roles and missions of the other participants, as well as time for formal or informal exchanges, to guarantee the quality of the teamwork. According to some authors, ‘presenting one’s specific expertise and recognising the contribution of others also means realising with humility and openness the richness as well as the limits and uncertainties of one’s specific profession’ [25]. Some practices are specific to one’s professional category (e.g. physical care), while others are common to several, as if each person had a specific role in achieving a common objective. For example, anticipation was cited by all participants (symptoms to come, steps to take, possible psychological developments) (P21, P30 and P34), as was the use of a genosociogram [7] (P50). Each participant was thus given a place in the group with ‘the mission of ensuring the continuity of the group to which he belongs’ [26]. He or she adopts the values, ideals and culture of the group, in this case, the palliative culture. Thus, psychologists positioned themselves as a third party, analysing the dynamics at play among the various participants so as to be able to propose an interpretation of the situation [27] (P49). Nurses assumed a coordinating role between the possibility of ‘deferring non-urgent care when relatives are visiting’ and considering the patient’s socio-cultural and religious practices (P39 and P40), practices with a type of adaptability that would not necessarily be transferable to all working contexts. Physicians, who played a protective role [28], mentioned reassuring relatives and their increased involvement in requests for euthanasia from relatives (P16 and P34).

The vast majority of the practices reported were consensual (92%); there was no evidence of any circumstance that would lead to ‘words in tension’ or endanger ‘the fruitful bond of living together’ [29], and disagreements and conflicts that undoubtedly occurred were not recorded [30]. This supports moving in the direction of establishing a coherent practice. Sharing these practices with other PCU teams would make it possible to check whether they are common among PCUs, or whether this coherence depends on the palliative culture of the PCU.

### Caregiving

The care and comfort of the patient and his or her relatives are major elements of the support offered during the agonal phase. The care given to the patient was considered an accompaniment for the relatives, who were described by the professional caregivers to be ‘at the heart of suffering’ (P3). This support also involved attention to the physical well-being of the relatives, with a view to physiological balance (P4) and care of the body; massages and relaxation, through their containing function, offered security, allowing relatives to ‘free themselves emotionally’ [31] (P6 and P7). Care-assistants spoke of a ‘benevolent practice’, described in the literature as ‘therapeutic hugging’ and physical restraint [32], a benevolent practice of listening [33], which may be practised as part of the emotional or non-verbal communication between caregivers and realtives to which all are sensitive [34].

Relatives participate in some care or ‘co-care’ (care given with relatives) [35], a practice whose value has been studied [36]. Some authors have proposed that the relationship between professional caregivers and relatives in cancerology is ‘dialectical to say the least, if not [representative of] rivalry in the distribution of roles’ [37], which may put relatives at a greater or lesser distance [38]. However, in the PCU, co-care was offered to relatives to give them the possibility of (re) finding a role relative to the patient and to facilitate communication through touch.

Physicians listed a large number of practices related to the theme ‘Communicating, informing and explaining’, which reflects their particular involvement in interviews announcing the entry to the agonal phase. Considerable informations and explanations are provided to relatives to prevent possible misunderstandings. Some of this information concerned the evolution of the agonal phase, especially symptoms that have appeared or will appear in the future. At the same time, the professional caregivers explained the care provided to the patient and its usefulness for the patient’s comfort.

Relatives were very sensitive to the possibility of bidding farewell to the patient and being present at the time of death [34]. They demanded information on the disease and its future course; hence, the interest in proposing interviews [39]. Psychologists and physicians insisted that various topics be discussed during interviews with relatives (P16). Interviews with relatives helped people to get to know one another and reduced the gap between the reality of the situation and the perceptions of relatives. The interview may be expanded to include several relatives and may be conducted by several professionals in pairs or trios. On this occasion, relatives sometimes allowed themselves to express personal feelings (shame, guilt, injustice). They dealt with family dynamics, death, the future and more. Professionals described their intention to help relatives to ‘release guilt’ and ‘better understand the attitude of those around them’. According to the definition of the helping relationship, the caregiver will then have to ‘adapt his or her interventions according to his or her observations and offer support adapted to the person being cared for’ [40], while being aware of the need to ‘respect the pace of integration of information from relativesand to ‘question them about what is at stake for them’ at this stage of the illness. Tarberg et al. stressed that ‘a family perspective should be included in the concept of patient-centred care’ [39]. However, in this study, relatives were considered participants involved in the situation. Listening to relatives with a non-judgmental attitude enabled them to lay down their burden and provided them with genuine relief.

The moment when relatives enter the room was highlighted in these findings. Nurses mentioned that they provided care before the relatives entered and warned them of the medical devices and equipment they would find there (P1 and P17). Doctors underlined the psychological preparation necessary before relatives entered the room. These practices reflect the need to anticipate the meeting between relatives and the patient because the patient has potentially changed physically since the last visit, or because death has occurred. These practices aim to reduce the possible shock and facilitate physical approach of the dying person 

A study suggests that certain bereavement experiences and support needs are specific to family caregivers providing end-of-life care, although this remains a poorly investigated issue. This paper focuses on themes related to bereavement, which were derived from an analysis of free text survey responses (1403 patients, carers, professionals, volunteers and members of the public). The responses demonstrated relationships among death experiences, feelings of guilt, and bereavement outcomes for some family caregivers, as well as caregiver experience of a ‘void’ created by the withdrawal of professional support after death [41].

Thus, considering relatives as caregivers is important, and helping them to help is just as important. This ‘complex and frequently reversible set of transactions between those who help and those who are helped’ [42] is called caregiving, which, in a context of dependency, underlies the involvement of caregivers and support volunteers.

### Evocation of death and the wish to hasten death

Approaching death was evoked by all speakers during the agonal phase. Despite lack of knowledge about this phase, professionals seemed to have some clinical understanding of the agonal period and its symptoms, which they could describe and explain, while noting the limits of their knowledge about this period of life (P17–P25). This capacity for observation goes hand-in-hand with the quality of care and is itself a guarantee of quality support for relatives. Interviews announcing the proximity of death were conducted by a physician, often with a colleague (physician/psychologist or physician/nurse pair) (P20 and P22). Non-medical listening times, considered ‘human times’ for dialogue and sharing, seemed just as important to relatives (P31 and P35–P38). They were able to express themselves and to elaborate and reformulate the information received with the accompanying person. This process echoes Bion’s theory on the interaction between the baby’s psyche and the maternal psyche, considering that through the alpha function, ‘impressions of the senses and emotions’ are no longer felt by the baby ‘as things in themselves, undigested facts, raw experiences’, but will become ‘elements available for thought’ [43]. The death of another can then be considered.

Accompanying the patients’ relatives at the end of life can influence the course of the relatives’ mourning [2, 6, 40] and prevent psychiatric comorbidities after the patient’s death [44]. This reality underpins communication among carers, which is essential to their ability to be of help in all its clinical, practical, psychosocial and spiritual dimensions [45].

The practices reported by professional caregivers tended to allow relatives to remain present with the patient in a new way and to prepare them for that person’s disappearance. Tending to them (e.g. offering coffee) (P5), listening to them, proposing that they stay in the unit (P42), inviting them to take meals there, taking time for them (P9), providing a massage (P6), having helpful talks and providing psychological support (P16) are all part of the preparation for mourning and for a future despite absence. In addition, interveners can be attentive to certain factors that complicate grief, such as the relationship between the patient and a relative, the age of a relative, his or her health, professional status and the repetition of grief.

Finally, 12 practices cited during FGs mainly by physicians concerned the relatives’ requests for euthanasia (P16 and P34). Studies have primarily considered the ‘wish to hasten death’ as expressed by the patients themselves [46–48]. The authors emphasise the value of questioning the origins of this wish and its meaning and function for the patient in all its forms, ranging from a hypothetical consideration of hastening death to an explicit request [49]. Physicians were particularly confronted with this sometimes-repeated request. They proposed elaborating on this request and on what might generate it, such as ambivalence, feelings of guilt, the experience that death is ‘unbearable’, etc. The literature also suggests that in these cases, carers experience a feeling of powerlessness [50], calling into question their skills and the institution’s capabilities.

Some relatives felt a lack of availability from the carers and a feeling of loneliness after the patient died; some mentioned that the support they received was not entirely satisfactory. According to some authors, a sufficiently good team is one that is ‘devoted, rigorous, meeting limits and concerned about them’ [25]. A good enough intervener would be one who tries to meet the needs of relatives in this period, knowing that they will not be able to avoid certain types of grief (loss of control and communication) and frustration.

## Conclusions

This exploratory study generated a list of support practices deemed desirable by an interprofessional PCU team, palliative-care volunteers and relatives. This study has certain limitations. First, the number of participants was limited. It was not possible to bring together all professional caregivers across professions in the middle of the day (with the exception of physicians). Only one FG was conducted per category, and it lasted 1–2 h, depending on the group. The number of practices mentioned might have depended on the number of professionals present and their comfort with speaking in groups. The second limitation concerns the non-exhaustive quality of the practices listed. Despite the effort made to report the results to all professionals in the unit and to elicit their feedback, this list cannot be considered exhaustive.

This preliminary work was nevertheless necessary as the basis for a second, broader study that would seek to establish a consensus on these practices through a broad survey of professionals and experts working in PCUs and other palliative-care contexts using an adapted methodology. It is important to remember that even though observing a loved one in the agonal phase is among the most painful trials one may encounter, no recommendations concerning support for relatives have been offered to date. There is therefore a definite need for further research regarding this issue.

Classifying support practices for the relatives of patients in the agonal phase will make it possible to develop training modules for teams working with relatives, in institutions or in the home. Given their participation in the development of practices relevant to the agonal phase, the meaning that caregivers assign to their work could contribute to improving their quality of life. Future studies should aim to improve support for the relatives of patients in the agonal phase, as well as promote the well-being of patients and their relatives. If patients and their relatives are better supported during the agonal phase, the experience of this currently dreaded phase of life may eventually improve.

Studies on the impact of this support on the quality of life of relatives and the experience of bereavement (which can be classified as “normal”, “complicated”, or “pathological”), as well as the medical, economic and social consequences, would also be valuable.

## Supplementary Information

**Additional file 1.** Interview guide FG professionals and support volunteers.

**Additional file 2.** Interview guide relatives.

**Additional file 3.** List of practices with the provider(s) of each practice.

**Additional file 4.** Coreq Check list.

## Data Availability

The datasets used and/or analysed during the current study are available from the corresponding author on reasonable request.

## Abbreviations

PCU: Palliative care unit
WHO: World Health Organization
IPs: Individual professionals
FGs: Focus groups
P: Practices
N: Nurses
CA: Care-assistant
PHY: Physician
PSY: Psychologist
PSYCHOMET: Psychometrician
SECRET: Secretary
SEA: Socio-educative assistant
SOCIOAESTH: Socio-aesthetician
HE: Health executive
PHYSIO: Physiotherapist
HAS: Hospital service agent
PCV: Palliative-care volunteer
REL: Relative
