# Supplementary material for: Support practices by an interdisciplinary team in a palliative-care unit for relatives of patients in agonal phase
Source: BMC Palliat Care. 2020 Nov 19;19:173. doi: 10.1186/s12904-020-00680-4 (PMC7678093; doi:10.1186/s12904-020-00680-4)
Supplement: Supplementary file 1 — Additional file 1. Interview guide FG professionals and support volunteers. [file 12904_2020_680_MOESM1_ESM.docx]

**Interview guide FG professionals and support volunteers**

**Question 1**

What comes to mind about the relatives of a patient in agonal phase?

Reminders:

Attitudes/Behaviors observed in relatives

Questions from relatives

Expectations/Needs of Relatives

Emotions observed in relatives

Consensus building :

The most problematic situation

Most frequent situation

**Question 2**

What are your practices in place as ... (name the profession) with relatives during the agonal phase at PCU?

Reminders:

Why?

When?

(Examples: Explanations given to relatives, helping relationship with relatives.)

In relation to each practice, ask the rest of the group: "Is this a practice you have? »

**Question 3**Are there any practices that you have not mentioned, that you have never initiated (for lack of resources, training, etc.), but which you think are interesting to develop?


Let the participants express themselves and classify on flipchart:
- Consensual practices
o Current
o Occasional
o Expected (Practices that we do not have the means to carry out but that we would like to do)

- Non-consensual practices (more personal)
