# Supplementary material for: Support practices by an interdisciplinary team in a palliative-care unit for relatives of patients in agonal phase
Source: BMC Palliat Care. 2020 Nov 19;19:173. doi: 10.1186/s12904-020-00680-4 (PMC7678093; doi:10.1186/s12904-020-00680-4)
Supplement: Supplementary file 2 — Additional file 2. Interview guide relatives. [file 12904_2020_680_MOESM2_ESM.docx]

**Interview guide FG relatives**

Question 1

You have accompanied your loved one to the Palliative Care Unit of Saint André Hospital :

Concerning the last moments called agonal phase, what comes to your mind about the way you were accompanied by the team?

Question 2

What were your greatest difficulties?

Question 3

What did you mainly expect from the health care team?

Question 4

What practices were most appropriate for you?

Interviews

Attentions

Explanations

Outdoor relay

Offer to stay on site

Question 5

What was missing?

Searching for avenues of consensus: Filing on flipchart :

Consensus: Expectations

Practices carried out

Missing practices

Non-consensus: Expectations

Practices carried out

Missing practices
