# Supplementary material for: Support practices by an interdisciplinary team in a palliative-care unit for relatives of patients in agonal phase
Source: BMC Palliat Care. 2020 Nov 19;19:173. doi: 10.1186/s12904-020-00680-4 (PMC7678093; doi:10.1186/s12904-020-00680-4)
Supplement: Supplementary file 3 — Additional file 3. List of practices with the provider(s) of each practice. [file 12904_2020_680_MOESM3_ESM.docx]

**List of practices with the provider(s) of each practice in square brackets**

Abbreviations used below: N, nurses; CA, care-assistants; PHY, physicians; PSY, psychologists; PSYCHOMET, psychometrician; SECRET, secretary; SEA, Socio-educative assistant, SOCIOAESTH, socio-aesthetician; HE, Health executive; PHYSIO, physiotherapist; HAS, hospital service agent; PCV, palliative-care volunteer; REL, relative

**PROVIDING CARE, ENSURING COMFORT**

1. Provide patient care before the relatives enter the room, if necessary [N]

2. Support requests for relatives to perform care or care-sharing (co-care) for the patient, depending on the relatives (security, reassurance, investment, feeling of usefulness, desire, capacity) [N, CA, PHYSIO, PHY, REL]

3. Take care of relatives through care given to the patient (washing, perfuming, hairdressing, speech, music, make-up, manicure, mobilisation) [CA, PSY, PHYSIO, SOCIOAESTH, REL]

4. Attend to physical needs (drink, eat, sleep) [PHY]

5. Ensure the comfort of relatives in the unit (offer coffee, wine, breakfast) [CA]

6. Propose a massage to relatives if trained to do so [N, PSYCHOMET, SOCIOAESTH, PHYSIO, REL]

7. Propose an approach using relaxation, hypnosis, or eye movement desensitisation and reprocessing (EMDR), depending on the situation and the psychologist’s training [PSY]

8. Invite relatives to leave the room or to use the family room [N, PHY, PCV, REL]

9. Offer to provide some respite time for the family [N, CA, PHY, REL]

10. Allow family and friends to recreate a moment of intimacy with the sick person [CA, PSY]

11. Psychologically prepare relatives for their entry into the room [PHY]

12. Inquire about absent relatives [N, PHY]

13. Ensure that relatives are surrounded and supported by an entourage [PHY]

14. Attend to children [PHY, PSY]

15. If necessary, grant a request for make-up for the patient after death [SOCIOAESTH]

16. Conduct assistance interviews [N, SEA, REL]. Personalise these in terms of objectives and content (anticipation of death, the future, mourning, family dynamics, representations, agony, death, integration of information, non-judgment of relatives, feelings of injustice, guilt, previous experiences) [N, PSY, PHY] and in case of a request for euthanasia (temporality, ambivalence, causes for the request, meaning) [PHY]

**COMMUNICATING, INFORMING, AND EXPLAINING**

17. Inform relatives about what they will see in the room before entering; explain the medical devices and equipment once inside accompanying them to the room [N]

18. Answer questions related to pain [PHY]

19. Explain the care, its impact on the patient's well-being, and its continuation [N, CA, PSY, SOCIOAESTH]

20. Announce entry into agonic phase [PHY, REL]

21. Help relatives to recognise the signs of agony that will appear [CA, PHY, REL]

22. Explain the patient's condition and visible symptoms [PHY]

23. Answer questions regarding the patient's level of awareness of reality [PHY]

24. Check whether the expectations of family members are being met [CA, PHY]

25. Inform relatives that caregivers will be entering the room more often because the patient can no longer call them [PHY, REL]

26. Inform of the imminence of death [N, PHY, REL]

27. Respect the relatives’ wishes concerning the announcement of the death [N]

28. For relatives who wish to be present at the time of death, warn them that this may not be possible [PHY]

29. Give relatives an opportunity to indicate that they do not wish to be present at the time of death [PHY]

30. Inform relatives that they can call whenever they want to, even at night [N, PHY]

31. Anticipate the steps that will need to be taken after death (clothing, family records, funeral, bereavement, and follow-up) [N, PSY, SEA, HAS, PCV]

32. Announce the death to relatives in person or by phone (time for silence, beginning the sentence), provided that the nurse has received formal or informal training [N]

33. Make physical contact with loved ones (touch or be touched) as the situation arises [CA, PSY, PCV, SEA]

34. Receive the request for euthanasia (reminder of the ethical and legal framework, advance directives, trusted person, sedation, objective of treatment) [PHY, CA]

35. Talk about something other than the situation [HAS, REL]

**INTERACTING**

36. Welcome and approach relatives [N, CA, PHY, SECR, SEA, HE, PCV]; speak to them in the corridor if they are not familiar [N]; show availability in a non-verbal way [PSY]; establish a climate of trust [HE]

37. Propose listening times (feelings, reformulations, silence), a silent presence [PCV]

38. Propose a formal interview [CA, PHY, PSY, SEA, SOCIOAESTH]; in a dedicated space [CA, PHY, PSY, HAS, PCV]; with others who are close to the patient [PHY, PSY, SEA, SOCIOAESTH, PCV]; include several professionals [PHY, PSY]; in person or by telephone [PSY]; post a sign to indicate that the room is in use [PSY]; schedule the interview outside regular hours if necessary [PSY]; especially in the case of a request for euthanasia [PHY]

39. Defer non-urgent care if a close relatives visits [N]

40. Consider the patient’s socio-cultural and religious practices [N]

41. Keep young children occupied during the visit [CA, PCV]

42. Propose that relatives stay the night [PHY, REL]

43. Encourage family and friends to contact the doctors and members of the care staff [PHY, REL]

**MOBILISING INTERDISCIPLINARY SKILLS**

44. Work in pairs such as nurse and nursing assistant [N, CA, SEA, SOCIOAESTH]

45. Propose a multi-professional interview [N, PSY, HE]

46. Specifically include attending to young children during an interview with other professionals present, including the psychologist [N, PHY, PSY].

47. Hand off tasks between peers [N, CA, PSY, SEA, HAS, PCV]

48. Pass the patient care role on to other members of the PCU and to cultural representatives (propose contacts to relatives and pass information on to colleagues) [N, CA, PHY, PSY, SECRET, SOCIOAESTH, SEA, HSA, PCV, REL]

49. Serve a third-party function between the team, family, and patient [PSY]

50. Design an interdisciplinary support project for relatives (particularly using a genosociogram) [N, PHY, PSY, SEA, HE, PCV]

51. Consider setting up a weekly meeting with relatives to discuss the general functioning of the PCU and to inform them of the team's position on certain issues (sedation, euthanasia, agony, nutrition, etc., plus explanation of PCU practices) with the participation of caregivers and palliative-care volunteers [PHY, PSY]

52. Provide talking spaces (transmission meeting, speaking group...) [HE]
